# A Protocol for Diagnosis and Management of Aortic Atherosclerosis in Cardiac Surgery Patients

**DOI:** 10.1155/2017/1874395

**Published:** 2017-08-09

**Authors:** Wouter W. Jansen Klomp, George J. Brandon Bravo Bruinsma, Arnoud W. J. Van 't Hof, Jan G. Grandjean, Arno P. Nierich

**Affiliations:** ^1^Department of Cardiology, Isala, Dokter van Heesweg 2, 8025 AB Zwolle, Netherlands; ^2^Department of Clinical Epidemiology, Julius Center for Health Sciences and Primary Care, University Medical Center Utrecht, P.O. Box 85500, 3508 GA Utrecht, Netherlands; ^3^Department of Cardiothoracic Surgery, Isala, Dokter van Heesweg 2, 8025 AB Zwolle, Netherlands; ^4^Department of Cardiology, Maastricht University Medical Center, P.O. Box 5800, 6202 AZ Maastricht, Netherlands; ^5^MIRA Institute for Biomedical Technology and Technical Medicine, University of Twente, P.O. Box 217, 7500 AE Enschede, Netherlands; ^6^Department of (Thoracic) Anesthesia and Intensive Care, Isala, Dokter van Heesweg 2, 8025 AB Zwolle, Netherlands

## Abstract

In patients undergoing cardiac surgery, use of perioperative screening for aortic atherosclerosis with modified TEE (A-View method) was associated with lower postoperative mortality, but not stroke, as compared to patients operated on without such screening. At the time of clinical implementation and validation, we did not yet standardize the indications for modified TEE and the changes in patient management in the presence of aortic atherosclerosis. Therefore, we designed a protocol, which combined the diagnosis of atherosclerosis of thoracic aorta and the subsequent considerations with respect to the intraoperative management and provides a systematic approach to reduce the risk of cerebral complications.

## 1. Introduction

Atherosclerosis of the upper thoracic aorta can be accurately visualized with modified transesophageal echocardiography (TEE A-View method). In patients undergoing cardiac surgery, use of perioperative screening for aortic atherosclerosis with modified TEE was associated with lower postoperative mortality, but not stroke, as compared to patients operated on without such screening [1]. The preprocedural focused TEE is part of the “Isala Safety Checklist,” which covers patient-specific items related to risk factors for complications in the perioperative period. The patient-specific risk factors are debated just before skin incision, since this is the moment that all information is up to date and complete. At this point, surgical strategy can still be adapted [2].

**Figure 1:**
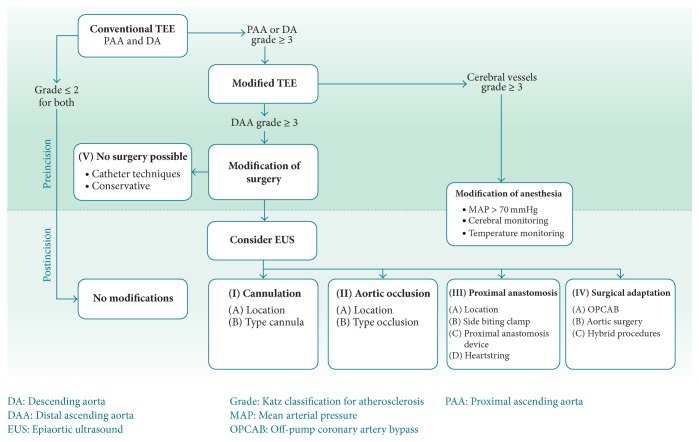
Flowchart of the diagnostic and therapeutic management of aortic atherosclerosis in cardiac surgery.

At the time of the study we did not yet describe the indications for modified TEE in a protocol and the changes in patient management in the presence of aortic atherosclerosis were not standardized. Therefore, we designed a protocol, which combined the diagnosis of atherosclerosis of thoracic aorta and the subsequent considerations with respect to the intraoperative management (Figure 1). This protocol consists of two parts, A: diagnosis of aortic atherosclerosis and B: operative management, and provides a systematic approach to reduce the risk of cerebral complications.

*Part A: Diagnosis of Aortic Atherosclerosis*. As recommended, transesophageal echocardiography should be performed in all patients at the start of surgery, that is, before sternotomy, to perform a complete cardiac interrogation, and to subsequently screen for atherosclerosis of the proximal ascending aorta and the descending aorta. No atherosclerosis of grade 3 or more of the descending aorta has a negative predictive value of 94% for the absence of severe atherosclerosis in the ascending aorta [3]. Therefore, if no severe atherosclerosis is visualized in these parts, the chance of atherosclerosis of the distal ascending aorta (DAA) is limited, and surgery can be continued as planned without further imaging of the DAA. If atherosclerosis grade 3 or greater is visualized in either part, additional imaging of the DAA, the aortic arch and (if possible) its branches should be performed, which is possible to perform by modified TEE before sternotomy. This innovative way of monitoring has been shown to accurately diagnose aortic atherosclerosis of the DAA [3–5]. Epiaortic ultrasound (EAU) imaging can be done after sternotomy (additional to modified TEE). With EAU, focused visualization of both the proximal and distal ascending aorta is possible. Although still not routinely applied in all settings, EAU might be considered to verify grade ≥ 3 atherosclerosis before altering the surgical strategy.

*Part B: Operative Management*. A complete investigation of the aorta before surgery provides important information about the atherosclerotic burden of the aorta and guides the surgical team in making appropriate decisions for the individual patient.

## 2. Surgical Modifications

Presence of severe atherosclerosis of the thoracic aorta should raise several questions regarding the optimal perioperative management. We summarized the possible modifications of the surgical and anesthesiological management if atherosclerosis of the aorta is present. These changes were depicted in the lower part of Figure 1 and can be categorized in the following groups:

- Cannulation.
- Aortic occlusion.
- Proximal anastomosis.
- Surgical adaptation.
- No surgery possible.

The figure aimed to provide a simple and comprehensive overview of the possible changes in the surgical management. In the respective sections below we provide further information for each subject with an overview of the prevailing literature.

*(I) Cannulation*. The DAA is typically preferred for cannulation since this is most easily accessible. A different location may be preferable in the presence of atherosclerosis or aneurysmal widening of the ascending aorta or aortic arch [6]. We will briefly describe the advantages and disadvantages of each alternative, which were also elaborately described in recent guidelines [6]. The eventual strategy should be weighted in each separate case, based on the severity and extent of aortic atherosclerosis of the ascending aorta, aortic arch and main cerebral vessels, type of cerebral protection, anatomical considerations, and surgeon's capabilities.

*(A) Location*. In many cases of localized atherosclerosis, simply moving the cannula away from atherosclerotic areas may suffice. Indeed, a different cannulation site is the most frequent change in the surgical technique if imaging shows atherosclerosis of the ascending aorta [7]. Multiple nonrandomized studies have suggested that the incidence of stroke can be reduced by avoiding manipulation of aortic atheroma during cannulation. A randomized study which compared a strategy with and without perioperative screening with epiaortic ultrasound did however not find a difference in perioperative embolization or postoperative neurological complications, despite a significant change in the positioning of cannulation [7].

*Femoral*. In patients with atherosclerosis of the ascending aorta, femoral cannulation has been a much used alternative to central cannulation [8]. The aim is to prevent stroke through the prevention of plaque mobilization in the ascending aorta. Several retrospective studies have questioned its use however, because of concerns that this technique may actually increase the incidence stroke because of flow reversal and the possible mobilization of the usually even more atherosclerotic descending aorta [9]. Although a more recent study did not confirm this supposed association between femoral cannulation and increased stroke or mortality, central cannulation may be preferable in the sclerotic aorta [8]. Femoral cannulation has an important place for individual patients, for example, in surgery for type A aortic dissection.

*Axillary or Subclavian*. Reports on the above-mentioned increased incidence of stroke after femoral cannulation led to an increasing use of axillary or innominate artery cannulation [8, 10, 11]. There are still some controversies regarding the potential lower risk with axillary or subclavian cannulation compared to femoral or direct aortic cannulation [7–9, 11]. According to recent guidelines on the surgical management of aortic valve and ascending aortic disease, axillary cannulation should be considered in patients with a calcified or porcelain aorta [6].

*(B) Type of Cannula*. The type of cannula is important since it directs the high flow during extracorporeal circulation into the aorta [12, 13]. Typically, aortic cannulation is performed with a straight cannula. After an angulated introduction in the anterior wall, the jet is directed at the posterior wall of the ascending aorta, which has been suggested to cause a “sand-blasting effect” which may release atherogenic emboli [14]. Also changes in shear stress caused by different aortic flow patterns have been recognized during cardiopulmonary bypass (CPB) and could explain the erosive effect of the high-velocity jet from aortic cannula [14, 15].

Therefore, other types of cannulas were designed, aimed to reduce the stress on the aortic wall. Bent-tip catheters should prevent a flow directed at the aortic wall; however they create a potential sand-blasting effect into the aortic arch and cerebral vessels [16]. The incidence of stroke was lower in patients with aortic cannulation using a bent-tip catheter (0.9 versus 1.8%); the authors did however not correct for differences in baseline risk between both groups [16]. Another type of aortic catheter (Select 3D® arterial cannula; Medtronic Inc., Minneapolis, MN) produces four jets with a reduced outflow velocity [12, 16, 17]. The tip is also curved, which together should reduce the shear stress on the aortic wall. No studies yet studied the impact of this catheter on the prevention of postoperative complications.

*(II) Aortic Occlusion*. The default procedure for aortic occlusion is the placement of an aortic cross-clamp. Other possibilities are endoballoon occlusion and hypothermic circulatory arrest.

*(A) Location*. During CPB, aortic cross-clamping is a normal procedure to allow cardioplegic solution to enter the coronary arteries to arrest the heart. The placement and release of a clamp are however associated with increased transcranial Doppler detected emboli, and may cause overt stroke. This was nicely demonstrated in a postmortem study, which associated the positioning of a cross-clamp with stroke [18]. They concluded that, before placement of a clamp on the aorta, echo evaluation is mandatory in order to reduce adverse events.

*(B) Type of Occlusion*

*Endoballoon Occlusion.* Placement and release of a traumatic aortic cross-clamp have been related to an increased incidence of cerebral emboli. In order to prevent this, it has been proposed that inflation of a balloon in the aorta is less traumatic and may thus reduce cerebral embolization. In a randomized comparison of CPB with endoclamping or transthoracic clamping in patients who underwent minimal invasive mitral valve replacement, the incidence of transcranial Doppler- (TCD-) measured solid emboli in the middle cerebral arteries was lower in the endovascular group when applying and releasing the clamp [19]. However, patients with aortic atherosclerosis were excluded from this study, while it would be interesting to know if the use of an endoballoon also reduces cerebral embolization when applied in a diseased aorta. It is unlikely that, in a diseased aorta, a sliding aortic balloon is a stroke risk-reducing procedure in robotic or minimally invasive cardiac surgery. Reports have described its use in severely atheromatous aortas where the risks of dislodgment after external cross-clamping or intermittent fibrillatory arrest were deemed too high [20]. Severe disease of the ascending aorta is however considered a relative contraindication for this endoaortic balloon. Placement of an endoaortic cross-clamp requires cannulation of the femoral artery or ascending aorta, both of which may cause dislodgement of atheroma itself.

*Hypothermic Circulatory Arrest and Cerebral Perfusion*. In the severely diseased aorta, replacement of the ascending aorta might be considered as a last resort treatment necessitating profound hypothermic circulatory arrest (PHCA) in order to perform the procedure. In a review by Lee and colleagues the use of antegrade- and retrograde cerebral perfusion (ACP and RCP, resp.) and profound hypothermic circulatory arrest were discussed [21]. Although many nonrandomized studies have reported outcomes associated with ACP, RCP, and PHCA, these are difficult to compare due to heterogeneity of study populations, and of techniques for cerebral protection and other aspects of the surgical procedure. The stroke rate after ascending aortic and aortic arch surgery using PHCA may be as low as 3.1%, with an increased risk if PHCA time exceeded 40 minutes (13.1%). One study randomized patients to PHCA or ACP and showed a higher jugular venous oxygen saturation in the ACP group, but no differences in neurological outcomes [22]. Three studies compared ACP and RCP [22–24], none of which showed a difference in the mortality or stroke rate, although ACP was associated with improved cerebral perfusion [23], and a lower incidence of (transient) neurologic deficits [24]. Since embolic complications rather than metabolic insufficiency cause stroke, it has been proposed that the beneficial hemodynamic properties of ACP are counterbalanced by a higher risk of cerebral emboli associated with the introduction of catheters in the arch vessels. Assessing these arteries before manipulation is a possible solution in order to reduce embolic load to the brain [21].

*(III) Proximal Anastomosis*. Total arterial or T-graft revascularization may obviate the need for a proximal anastomosis, coronary revascularization can thus be performed during no-touch OPCAB. Although theoretically this would reduce aortic manipulation and thus may prevent dislodgement of aortic atheroma, limited randomized studies have been performed to study this hypothesis. In a small prospective OPCAB study, it was determined whether a clampless facilitating device (CFD) performing proximal aortocoronary anastomoses would result in a lower incidence of cerebral embolic events compared with a partial clamping strategy during OPCAB. The study included 57 patients after screening the aorta by epiaortic ultrasound had confirmed mild aortic disease (grades I and II, i.e., plaques up till 3 mm). The patients were then randomly assigned to have proximal anastomoses using a partial-occluding clamp or a CFD. The endpoint of solid and gaseous emboli in the middle cerebral arteries was detected by using TCD. In these patients with a low quantity of aortic atherosclerosis, the use of a partial side biting clamp on the ascending aorta during OPCAB was associated with more cerebral embolic events compared with the patients treated with the CFD. Extrapolation of these results to daily practice is however limited since patients with plaques > 3 mm were excluded [25]. In a large cohort study of 4314 patients, both OPCAB and aortic no-touch technique reduce stroke after CABG. They evaluated the impact of partial aortic clamping versus a no-touch technique using either the Heartstring device or total arterial revascularization on the incidence of stroke. OPCAB was superior with regard to risk-adjusted outcomes, but only if no touch of the aorta was applied with a superiority of the CFD group. However, no information was provided about aortic disease as one of the major risk factor of perioperative stroke [26]. In a retrospective study including 160 patients aged >75 years who underwent OPCAB a no-touch strategy was compared to the use of a side biting clamp; the latter group had more neurological events or mortality (12.3 versus 2.9%, *p* = 0.035), which remained significant after correction for other variables [27]. A second study similarly identified partial aortic clamping as a risk factor for stroke after OPCAB.

*(IV) Surgical Adaptation*

*(A) Off-Pump Surgery*. Coronary artery bypass surgery on a beating heart precludes multiple of the previously mentioned risk factors for the development of postoperative neurological complications, that is, aortic cannulation, aortic cross-clamping, and antegrade cardioplegia, as well as the possible cerebral inflammatory response to CPB [27, 28]. In earlier randomized trials OPCAB was associated with reduced short-term postoperative cognitive dysfunction and a lower rate of bleeding, transfusion, and respiratory complications, an increased risk of early revascularization, and no effect on stroke, myocardial infarction, renal-failure, or death [29–31]. The largest randomized study to date, the Randomized On/Off Bypass (ROOBY) trial, showed however no benefit of OPCAB with an increased incidence of the primary composite endpoint (9.9% versus 7.4%, *p* = 0.04) and cardiac mortality (2.7 versus 1.3%, *p* = 0.03); also, graft patency was reduced in the OPCAB group (82.6 versus 87.8%, *p* < 0.01) [32]. A more recent meta-analysis showed that OPCAB was associated with a 30% (95% CI: 1–51%) relative risk reduction for stroke, in the absence of a difference in 30-day mortality or myocardial infarction [33]. This is an important finding, as the incidence of stroke is too low to expect single trials to show a difference in the stroke rate [33]. Given an absolute difference in postoperative stroke of 0.7% (2.1 minus 1.4%), the number needed to treat with OPCAB to prevent one stroke is approximately 143.

A flaw of these trials however was that the individual preoperative stroke risk was not used to select patients at a higher risk of developing stroke, for example, with an increased risk based on patient characteristics [34], or with preoperative visualization of the aorta to identify patients with aortic atherosclerosis. The reported trials did also not stratify their results for presence and absence of aortic atherosclerosis in a post hoc analysis, which would explain the limited effect on stroke reduction. Most studies, however, have not differentiated between clampless and off-pump techniques. Avoiding partial aortic clamping during OPCAB provided superior neurologic outcome and identified partial aortic clamping as the only independent predictor of stroke [26, 34]. In a comparison with percutaneous interventions, OPCAB with no-touch treatment showed the same low incidence of stroke of 0,8%, compared to on-pump CABG of 2,2% [35]. Also, in a recent meta-analysis comparing the risk of stroke in CABG versus OPCAB, off-pump surgery does not seem to reduce the risk of stroke. A pooled analysis of more than 4000 patients from 11 randomized clinical trials did not reveal any benefit of avoiding CPB on stroke risk. A possible explanation is that most OPCAB patients still undergo aortic manipulation because the side biting clamp is used for proximal anastomoses. This seems to be confirmed in the same meta-analysis that compares also bypass surgery with and without manipulation of the ascending aorta. It reports a spectacular reduction in stroke risk from 1.3 percent to 0.3 percent [36].

*(B) Aortic Surgery*. Replacement of the ascending aorta is recommended in the aneurysmal aorta (>45 mm), but guidelines do not give advice with regard to the optimal treatment of the severely sclerotic aorta. It can however be considered to prevent perioperative stroke, but also to prevent late strokes caused by mobile plaques. In a study by Zingone et al., a major change to the surgical procedure was deemed necessary after EAU evaluation in 152 of 1927 (7.9%) of patients who underwent cardiothoracic surgery, of whom 36 received a replacement of the ascending aorta [37]. Stroke was observed in one patient; two patients died before hospital discharge. Another study similarly reported the outcomes of 36 patients with AA-replacement because of intraoperative identification of AA atherosclerosis; one patient developed stroke and died during hospitalization [38]. In a third study, however, both the stroke rate (3/17) and mortality (4/17) were substantial [39]. Furthermore, aortic arch endarterectomy has been shown to be associated with an increased risk of postoperative stroke (OR 3.6, *p* = 0.001) in an already high incidence of stroke among patients with aortic atheroma (15.3%) [40].

*(C) Hybrid Procedures*. Alternatively, a hybrid procedure combining surgery and percutaneous intervention may be useful in selected patients, for example, with surgical revascularization using the left internal thoracic artery and subsequent percutaneous complete revascularization. However, passing the diseased aorta during cardiac catheterization carries a (limited) risk itself for cerebral complications [41].

*(V) No Surgery Possible*. The first aim of any treatment is “Primum non nocere.” In very unusual cases, the newly acquired information on the severity of aortic atherosclerosis may result in a reconsideration of the surgical procedure itself. After consideration of the previously addressed aspects of surgery, the risk of perioperative complications may be deemed too high to proceed. Alternative treatments should be considered, for example, a percutaneous coronary intervention for coronary artery disease, or percutaneous valve replacement for aortic stenosis.

## 3. Modification of Anesthesia

*(A) Perioperative Blood Pressure*. Invasive hemodynamic monitoring of blood pressure and central venous pressure belong to the basic armamentarium of monitoring during cardiac surgery. Not only embolization causes perioperative brain injury, but also hypoperfusion and subsequent ischemia; reperfusion injury plays a role [42]. Changes in intraoperative hemodynamic variables can be a cause for insufficient cerebral perfusion [43]. And patients with intracranial arterial stenosis are particularly vulnerable to variations in cerebral perfusion and are more prone for perioperative strokes. In patients with symptomatic cerebrovascular disease and preexisting ischemic symptoms, a stroke rate of up to 13% has been reported [44]. Several studies have shown that prolonged hypotension is a risk factor for adverse cerebral outcomes [44, 45]. Increased blood pressure in order to maintain adequate cerebral perfusion pressure should be implemented in case of cerebrovascular disease, unless cerebral monitoring depicts otherwise [43]. A threshold mean blood pressure of approximately 70 mmHg is necessary since studies in awake healthy subjects indicate that this is the lower limit of autoregulation, which is higher than the mostly recommended 50 mmHg from current textbooks [46]. In a randomized trial, Gold et al. showed that a higher mean arterial pressure (MAP; 80–100 mmHg) during bypass improves outcomes in long- and short-term follow-up when compared to low MAP (50–60 mmHg) [47].

*(B) Cerebral Monitoring*. Different types of cerebral monitoring devices such as bispectral index (BIS) and cerebral oximetry using near-infrared spectroscopy (NIRS) have been developed over the last decades in order to reduce cerebral morbidity related to the perioperative period. The BIS frequently applied a basic monitor for brain function in cardiac surgery, but the relation to improved neurologic outcome is still lacking. Independent of its direct application to awareness, the BIS information can be used to maintain an adequate balance between the anesthetic needs and the use of vasoactive therapies.

Cerebral oximetry using near-infrared spectroscopy allows measuring the saturation of brain tissue by a compilation of both venous and arterial blood within the brain. Although it is now already more than 20 years in clinical use, its beneficial effect on stroke and mortality reduction is not proven yet. One of the first studies using a NIRS treatment algorithm showed not an improvement in overall outcome but on the other hand a trend towards a reduction in stroke, only in patients who had an anesthetic tailored made interventional algorithm [47, 48].

Transcranial Doppler (TCD) may also identify stroke related manipulations during cardiac surgery and create the necessary awareness about the sources of embolization, such as emboli related to aortic manipulations or perfusionist interventions. The combination of different modalities such as TCD and NIRS together can identify different causes of cerebral hypoperfusion such as unrecognized cerebral venous obstruction, inadequate mean arterial pressure, or hypocapnic cerebral alkalosis. In a large, nonrandomized series of 1698 cardiac surgical patients reported by Goldman and colleagues, a significant reduction in perioperative stroke rate, from 2.0% to 1.0%, was observed in patients in whom rSO2 cerebral oximetry was used to optimize and maintain intraoperative cerebral oxygenation versus an untreated comparator group of 2077 similar patients operated on in the immediately preceding 18-month interval [49].

Using applied neuromonitoring techniques might result in improvement of patient outcomes and decrease of postoperative length of stay [50].

*(C) Temperature Monitoring*. The neuroprotective effect of cerebral hypothermia is assigned to decreasing cerebral metabolic rate and the effect protective cellular mechanisms including inhibiting excitatory neurotransmitter release [51].

Studies outlined the negative effect of hyperthermia during CPB on postoperative neurologic outcome; nevertheless no data are available showing a sustained benefit of cerebral hypothermia. Therefore is seems that cerebral hyperthermia is causing more brain damage than normothermia and strategies for perioperative temperature management are nowadays more focused on maintaining the normal physiologic borders [51, 52].

## 4. Limitations

**Figure 2:**
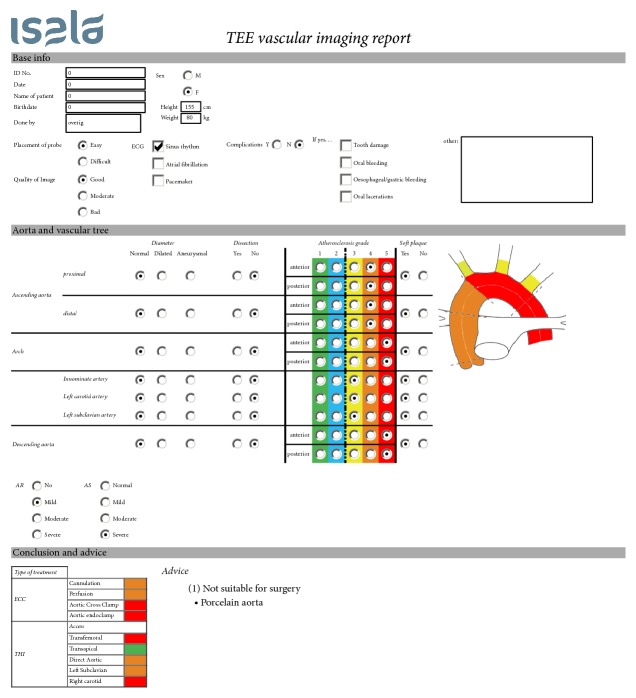
Example of a vascular report in the preoperative screening for possible aortic valve replacement in a patient with severe aortic stenosis. (Modified) TEE showed extensive calcifications of the complete thoracic aorta; it was therefore concluded to perform a transapical aortic valve replacement to minimize manipulation of the aorta.

The evidence for the efficacy of the individual changes in the surgical management is mostly limited to observational studies of limited size, except for OPCAB, which has been intensively compared to revascularization during CPB in multiple randomized trials. The absence of evidence should however not be a reason to ignore the well-known risks. We hope that this systematic approach to the diagnosis and management of aortic atherosclerosis can result in a reduction of postoperative cerebral complications. Future studies should focus on the effect of the implementation of this protocol on chances in the surgical management and on patient outcomes. For this purpose, we designed a form to uniformly register the degree of aortic atherosclerosis and the subsequent changes in the surgical management (Figure 2).

## 5. Conclusions

Neurological complications associated with cardiac surgery have been a primary concern. Many risk factors for stroke after cardiac surgery are known, of which atherosclerosis of the ascending aorta is of major interest. The stroke risk during cardiac surgery depends on the location and extent of disease, and on the amount of aortic manipulation. Routine use of intraoperative monitoring with (modified) transesophageal and/or epiaortic ultrasound should be applied in all cardiac surgery procedures in order to identify the patients at risk. The acquired diagnostic information should be used to tailor surgical strategies. We propose a systematic approach to identify aortic atherosclerosis and consider changes in the surgical perioperative management. These include aortic cannulation, aortic occlusion, proximal anastomosis, and surgical adaptation. Also modifications of anesthesia should be considered. Finally, in unusual cases a reappraisal of surgery itself may be appropriate, and percutaneous treatments should be considered. Although none of the described changes in the surgical management have unequivocal evidence of its efficacy, we hope that a holistic approach to this challenge will result in a reduction of stroke and other embolic related complications.
